# Upper gastrointestinal endoscopic findings in functional constipation and irritable bowel syndrome diagnosed using the Rome IV criteria: a cross-sectional survey during a medical check-up in Japan

**DOI:** 10.1186/s12876-023-02784-6

**Published:** 2023-05-03

**Authors:** Koji Otani, Toshio Watanabe, Kanae Takahashi, Yuji Nadatani, Masaki Ominami, Shusei Fukunaga, Shuhei Hosomi, Noriko Kamata, Fumio Tanaka, Yasuaki Nagami, Koichi Taira, Tatsuo Kimura, Shinya Fukumoto, Yasuhiro Fujiwara

**Affiliations:** 1 Department of Gastroenterology, Osaka Metropolitan University Graduate School of Medicine, 10/F, 1-4-3 Asahimachi, Abeno-Ku, Osaka, 545-8585 Japan; 2 Department of Premier Preventive Medicine, Osaka Metropolitan University Graduate School of Medicine, 12/F, 1-4-3 Asahimachi, Abeno-Ku, Osaka, 545-8585 Japan; 3 Department of Biostatistics, Hyogo Medical University, 1-1 Mukogawacho, Nishinomiya, Hyogo 663-8131 Japan

**Keywords:** Rome IV, Disorders of gut–brain interaction, Functional constipation, Irritable bowel syndrome, Kyoto Classification of Gastritis

## Abstract

**Background:**

The Rome IV criteria have been established as an international standard for diagnosing disorders of gut–brain interaction. In this study, we aimed to examine the upper gastrointestinal (GI) endoscopic findings and symptoms of subjects with functional constipation (FC) and irritable bowel syndrome (IBS) of individuals undergoing a medical check-up.

**Methods:**

A total of 13,729 subjects underwent a medical check-up at Osaka City University-affiliated clinic, MedCity21, between April 2018 and March 2019. Among the 5,840 subjects who underwent screening upper GI endoscopy and completed a questionnaire based on the Rome IV criteria, 5,402 subjects were consecutively enrolled after excluding subjects with a large amount of gastric residue (*n* = 6), those who had previously undergone partial or total gastrectomy (*n* = 40), or those with daily use of low-dose aspirin (*n* = 82), nonsteroidal anti-inflammatory drugs (*n* = 63), or acid secretion inhibitors (*n* = 308).

**Results:**

Robust Poisson regression analyses adjusted for age, sex, *Helicobacter pylori* infection status, alcohol intake, and smoking habits showed a significant association between FC and corpus erosion (adjusted prevalence ratio [aPR], 2.93; 95% confidence interval [CI], 1.51–5.67; *p* < 0.01) and red streaks (aPR, 3.83; 95% CI, 2.53–5.79; *p* < 0.01), whereas IBS was significantly associated with erosive gastritis (aPR, 8.46; 95% CI, 4.89–14.67; *p* < 0.01) and duodenitis (aPR, 7.28; 95% CI, 3.64–14.59; *p* < 0.01). Red streaks tended to be associated with IBS (aPR, 1.96; 95% CI, 1.00–3.83; *p* = 0.05). Subjects with IBS were the most to complain of both upper and lower GI symptoms and psychological symptoms, followed by those with FC and controls. IBS subjects with erosive gastritis or duodenitis had significantly more complaints of stomachache and feeling stressed than those without erosive gastritis or duodenitis (54.5% vs. 18.8%; *p* = 0.03 and 66.7% vs. 25.0%; *p* = 0.01).

**Conclusions:**

Subjects with FC and IBS had a variety of upper GI and psychological symptoms. In the upper GI endoscopic findings, corpus erosion and red streaks were associated with FC, and erosive gastritis, duodenitis, and possibly red streaks were associated with IBS.

## Background

The Rome IV criteria were established in 2016 as an international standard for diagnosing functional gastrointestinal (GI) disorders, which were recently referred to as disorders of gut–brain interaction (DGBI) [1–3]. DGBI is a group of disorders classified by GI symptoms related to a combination of various pathophysiology, such as motility disturbance, visceral hypersensitivity, altered mucosal and immune function, altered gut microbiota, and altered central nervous system processing [4, 5]. Among the DGBI, functional constipation (FC) and irritable bowel syndrome (IBS) are two major categories representing bowel disorders. FC is a bowel disorder in which symptoms of difficult, infrequent, or incomplete defecation predominate, and subjects with FC should not meet the diagnostic criteria for IBS [6]. Meanwhile, IBS is a bowel disorder that leads to recurrent abdominal pain associated with defecation or changes in bowel habits [6]. Although it is known that subjects with IBS frequently have upper GI symptoms [7], few studies have examined the upper GI endoscopic findings in subjects with FC and IBS as both are considered to be associated with the lower GI tract.

Functional dyspepsia (FD) is a gastroduodenal disorder that is characterized by symptoms of postprandial fullness, early satiation, epigastric pain, and epigastric burning associated with the upper GI tract [8]. There have been some reports on the upper GI endoscopic findings in subjects with FD. Chen et al. reported that FD subjects with gastric red streaks exhibited increased somatization and more stressful life events [9], and we previously reported that gastric depressive erosion is more likely to be found in subjects with FD than in healthy controls during a medical check-up [10]. Red streaks, which were originally named Kammrötung in German and has been called variously, such as comb-like redness or linear redness, can be seen from the lesser curvature to the greater curvature of antrum and corpus, and the degree of redness differs depending on the cases. According to the Kyoto Classification of Gastritis that was proposed in 2014 to standardize the upper GI endoscopic diagnosis of gastritis in Japan [11], red streaks are relatively common in *Helicobacter pylori*-uninfected mucosa, but can also be observed after *H. pylori* eradication and sometimes in *H. pylori*-infected mucosa. Depressive erosion is one of the erosive gastritis and can be observed in *H. pylori*-infected and uninfected mucosa and after *H. pylori* eradication. Although it has been generally considered that red streaks and depressive erosion are gastric acid-related lesions, they may appear with GI dysfunction. It is possible that they are also associated with other functional GI disorders, known as DGBI.

Therefore, in this study, we aimed to examine the upper GI endoscopic findings and symptoms in subjects with FC and IBS who were diagnosed using a questionnaire based on the Rome IV criteria in a large-scale survey during a medical check-up.

## Methods

### Study design

This was a single-center, cross-sectional survey of prospective participants.

### Study population

**Fig. 1:**
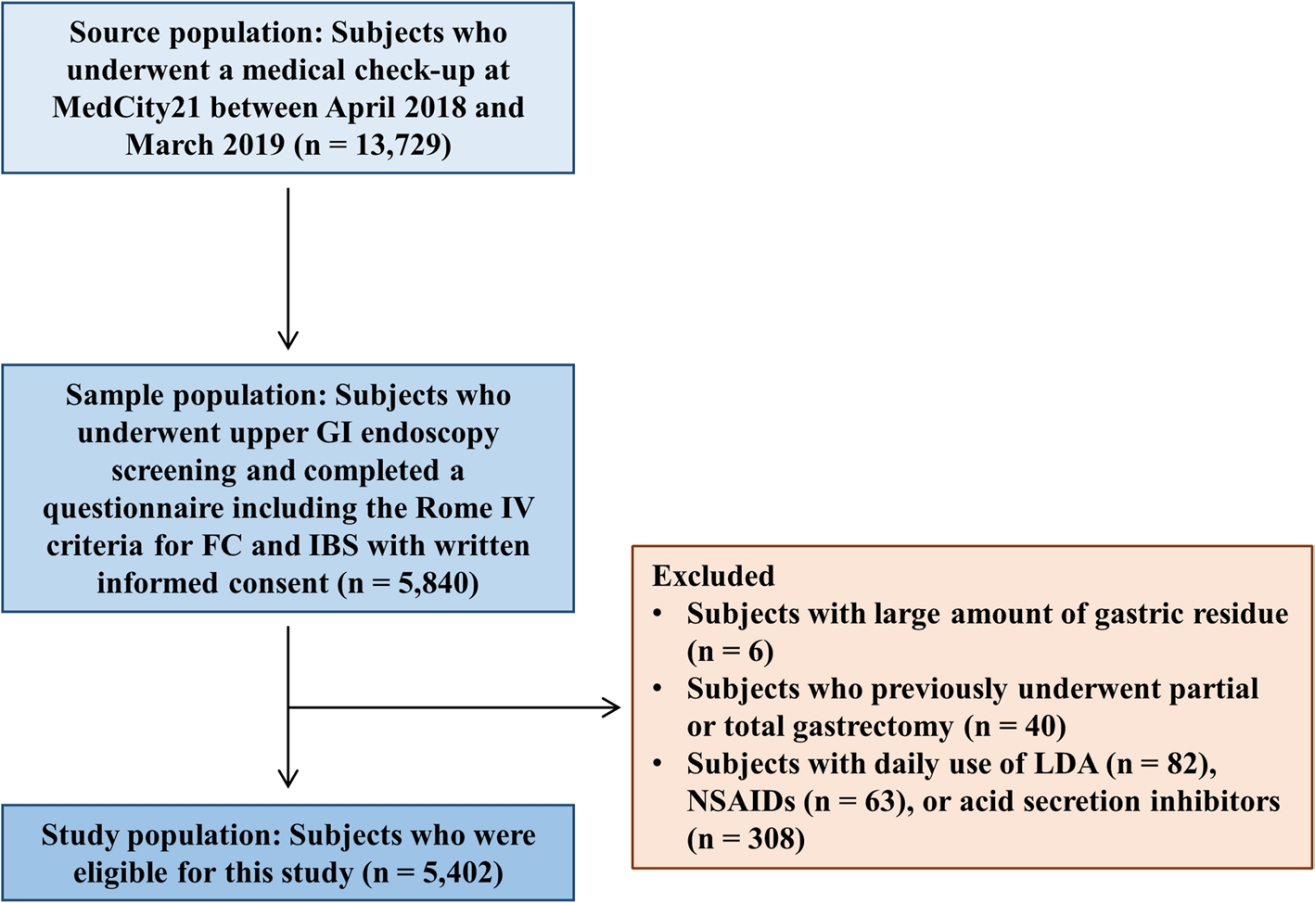
Schematic diagram of the study flow. Abbreviations: GI, gastrointestinal; FC, functional constipation; IBS, irritable bowel syndrome; LDA, low-dose aspirin; NSAIDs, nonsteroidal anti-inflammatory drugs

A total of 13,729 subjects underwent a medical check-up at Osaka City University-affiliated clinic, MedCity21, which provides medical examinations and health screening from the perspective of preventive medicine, between April 2018 and March 2019. Among the 5,840 subjects who underwent upper GI endoscopy screening and completed a questionnaire including the Rome IV criteria for FC and IBS with written informed consent, 5,402 subjects were consecutively enrolled after excluding subjects with a large amount of gastric residue (*n* = 6), those who had previously undergone partial or total gastrectomy (*n* = 40), or those with daily use of low-dose aspirin (LDA) (*n* = 82), nonsteroidal anti-inflammatory drugs (NSAIDs) (*n* = 63), or acid secretion inhibitors (potassium-competitive acid blockers, proton pump inhibitors, or histamine H2 receptor antagonists) (*n* = 308) (Fig. 1). None of the participants had advanced cancer. The clinical data of the participants were obtained from their medical records and general questionnaires that inquired about lifestyle habits and symptoms during a medical check-up.

### Diagnosis of functional constipation and irritable bowel syndrome

FC was diagnosed based on the Rome IV criteria, as follows: 1) the presence of more than two of the following: straining, lumpy or hard stools (Bristol Stool Form Scale type 1 or 2), sensation of incomplete evacuation, sensation of anorectal obstruction/blockage, manual maneuvers to facilitate defecation during more than one-fourth of defecation episodes, or fewer than three spontaneous bowel movements per week; 2) loose stools rarely present without the use of laxatives; 3) insufficient criteria for IBS; and 4) fulfillment of the criteria for the last 3 months with symptom onset at least 6 months prior.

IBS was diagnosed based on the Rome IV criteria, as follows: 1) recurrent abdominal pain occurring, on average, at least 1 day per week for the last 3 months and associated with more than two of the following: abdominal pain related to defecation, abdominal pain associated with a change in stool frequency, or abdominal pain associated with a change in stool form (appearance), and 2) fulfillment of the criteria for the last 3 months with symptom onset at least 6 months prior.

### Examinations of* H. pylori* infection

We used the anti-*H. pylori* antibody for serological diagnosis of *H. pylori* infection (E plate ‘Eiken’ *H. pylori* antibody; Eiken Chemical Co., Ltd., Tochigi, Japan). Information on *H. pylori* eradication history of subjects was obtained from their medical records or directly from them during a medical check-up.

### Upper gastrointestinal endoscopic findings

Screening with upper GI endoscopy was performed by several expert endoscopists using a 5.4 mm-diameter endoscope for upper GI endoscopy (GIF-XP290N; Olympus Medical Systems Co. Ltd., Tokyo, Japan). The endoscopic procedure was performed without sedation, and esophagus, stomach, and duodenum were observed using white-light imaging with indigo carmine as needed. The endoscopic findings were retrospectively double-checked and determined by one experienced endoscopist with more than 20,000 cases of interpretation to reduce inter-observer variance.

We used the Kimura-Takemoto Classification to evaluate the progress of atrophic gastritis [12]. If the atrophic border did not reach the cardia, the atrophic pattern was classified as closed-type. When the atrophic border extended beyond the cardia, it was classified as open-type.

**Fig. 2:**
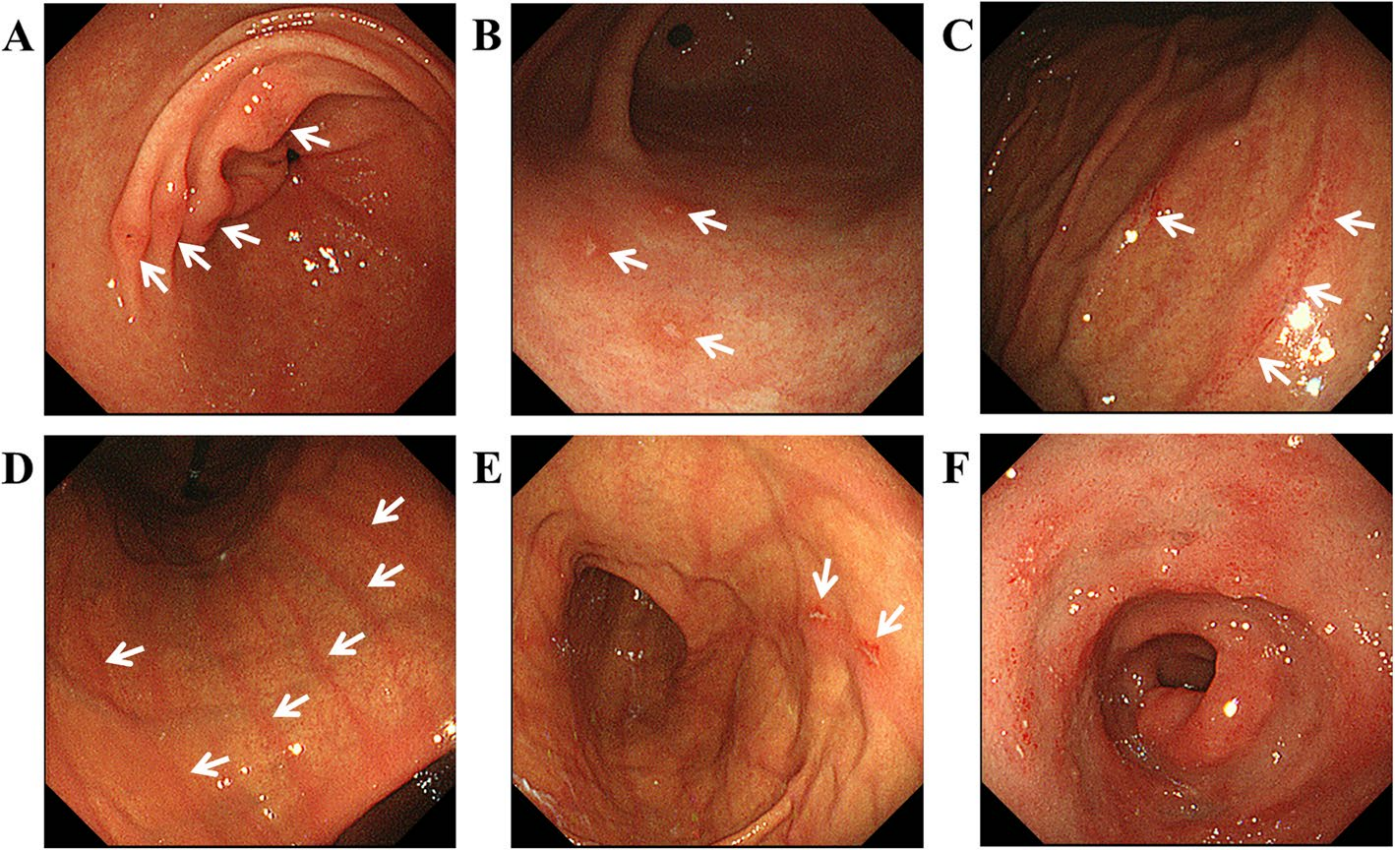
Typical endoscopic images of erosive gastritis, red streaks, and duodenitis. **A** Raised erosion, **B** Depressive erosion, **C** Corpus erosion, **D** Red streaks, **E** Corpus erosion on red streaks, **F** Duodenitis

Erosive gastritis is gastritis with tissue loss in the mucosal layer that penetrates no deeper than the lamina muscularis mucosae (Ul-I). According to the Kyoto Classification of Gastritis, erosive gastritis is further classified into raised erosion, depressive erosion, and corpus erosion. Raised erosion, also known as verrucous gastritis, is an elevated lesion with a shallow central depression that resembles an octopus sucker (Fig. 2A). Depressive erosion is a lesion in which the epithelium is deficient and the epithelial cells around the deficient area are flat (Fig. 2B). In this study, we defined raised erosion and depressive erosion as lesions located in the antrum, to be distinguished from the following corpus erosion. Corpus erosion includes longitudinal or linear depressive erosion or raised erosion located on the ridge of a fold or an extension line in the corpus (Fig. 2C, E). Red streaks are red lines on the mucosal surface that run longitudinally along the crests of the fold almost in parallel (Fig. 2D, E). Duodenitis is an inflammatory change in the duodenal mucosa and spotty, patchy, or band-like redness or erosion without ulceration can be observed (Fig. 2F).

### Objectives

The primary objective of this study was to examine the upper GI endoscopic findings associated with FC or IBS. The secondary objectives were the frequency of GI and psychological symptoms in subjects with FC, those with IBS, and controls and the relationship between symptoms and upper GI endoscopic findings associated with FC or IBS.

### Statistical analysis

Data are expressed as median and interquartile range (IQR) for continuous variables and as numbers and percentages for categorical variables. Robust Poisson regression analysis was performed to identify the upper GI endoscopic findings associated with FC or IBS, and the prevalence ratio and 95% confidence interval (CI) were estimated for each variable. The E-value represents the minimum strength for an unmeasured confounder that was associated with both the variables and the outcome, above and beyond the measured confounders, to explain away the association of the variables with the outcome [13]. E-values were calculated using the online E-value calculator (https://www.evalue-calculator.com/) [14]. Symptoms were compared using Pearson’s chi-square test or Fisher’s exact test to compare symptoms between two groups. Statistical calculations and analyses were performed using IBM SPSS Statistics Version 26 (IBM Corporation, Armonk, NY, USA) or R software, version 4.0.2 (The R Foundation for Statistical Computing, Vienna, Austria). *P*-values < 0.05 were considered statistically significant.

## Results

### Baseline characteristics of subjects with functional constipation, those with irritable bowel syndrome, and controls

The numbers of subjects who fulfilled the diagnostic criteria for FC and IBS were 108 and 49, respectively. The median ages of subjects with FC, IBS, and controls who had neither FC nor IBS were 51.0 (IQR, 44.0–59.0) years, 49.0 (IQR, 39.0–56.0) years, and 51.0 (IQR, 44.0–60.0) years, respectively. Females were the most frequent in subjects with FC, followed by those with IBS and controls (85.2%, 63.3%, and 50.4%, respectively). The median body mass index was lower in subjects with FC, followed by that in controls and subjects with IBS (21.0, IQR, 19.5–23.3; 22.3, IQR, 20.3–24.7; and 22.4, IQR, 20.0–25.2, respectively). There were no differences in *H. pylori* infection status among the subjects with FC, those with IBS, and controls.

Among the lifestyle habits in subjects with FC, drinking alcohol more than 5 days a week was the least common in subjects with FC, followed by that in those with IBS and controls (13.0%, 26.5%, and 26.7%). Exercising for ≥ 30 min at least twice a week for ≥ 1 year was the least common in subjects with FC, followed by that in those with IBS and controls (16.7%, 20.4%, and 26.6%, respectively). As for the lifestyle habits of subjects with IBS, getting enough rest by sleeping was the least common in subjects with IBS, followed by that in those with FC and controls (32.7%, 48.1%, and 59.3%, respectively). Subjects who skipped breakfast ≥ 3 times a week were more likely to have IBS, followed by controls and subjects with FC (26.5%, 13.7%, and 13.0%, respectively). Subjects who ate 2 h before going to bed ≥ 3 times a week were more likely to have IBS, followed by controls and subjects with FC (46.9%, 27.6%, and 22.2%, respectively) (Table 1).

**Table 1:** Baseline characteristics of subjects with functional constipation, those with irritable bowel syndrome, and controls

Variables	FC	IBS	Controls
No. of cases	108	49	5,245
Age (years, median [IQR])	51.0 [44.0–59.0]	49.0 [39.0–56.0]	51.0 [44.0–60.0]
Age group			
< 30	0 (0.0%)	1 (2.0%)	36 (0.7%)
30–39	11 (10.2%)	12 (24.5%)	557 (10.6%)
40–49	38 (35.2%)	13 (26.5%)	1,694 (32.3%)
50–59	34 (31.5%)	17 (34.7%)	1,568 (29.9%)
60–69	17 (15.7%)	4 (8.2%)	1,027 (19.6%)
≥ 70	8 (7.4%)	2 (4.1%)	363 (6.9%)
Sex			
Male	16 (14.8%)	18 (36.7%)	2,603 (49.6%)
Female	92 (85.2%)	31 (63.3%)	2,642 (50.4%)
BMI (kg/m^2^, median [IQR])	21.0 [19.5–23.3]	22.4 [20.0–25.2]	22.3 [20.3–24.7]
*H. pylori* infection status			
Anti-*H. pylori* antibody ≥ 10 U/mL without eradication therapy	3 (2.8%)	1 (2.0%)	180 (3.4%)
After *H. pylori* eradication therapy	26 (24.1%)	12 (24.5%)	1,249 (23.8%)
Others	79 (73.1%)	36 (73.5%)	3,816 (72.8%)
Alcohol intake			
≥ 5 days a week	14 (13.0%)	13 (26.5%)	1,399 (26.7%)
< 5 days a week	94 (87.0%)	36 (73.5%)	3,846 (73.3%)
Smoking habits			
Current smoker	11 (10.2%)	8 (16.3%)	743 (14.2%)
Never smoked or quit	97 (89.8%)	41 (83.7%)	4,502 (85.8%)
Exercise for ≥ 30 min at least twice a week for ≥ 1 year	18 (16.7%)	10 (20.4%)	1,393 (26.6%)
Walking (or an equal amount of exercise) for ≥ 1 h a day	39 (36.1%)	13 (26.5%)	2,135 (40.7%)
Walking faster than other people of the same age and sex	55 (50.9%)	22 (44.9%)	2,780 (53.0%)
Getting enough rest by sleeping	52 (48.1%)	16 (32.7%)	3,108 (59.3%)
Skipping breakfast ≥ 3 times a week	14 (13.0%)	13 (26.5%)	717 (13.7%)
Eating 2 h before going to bed ≥ 3 times a week	24 (22.2%)	23 (46.9%)	1,446 (27.6%)
Eating snacks and sweet drinks in addition to three meals in a day	93 (86.1%)	41 (83.7%)	4,090 (78.0%)
Eating faster than other people	47 (43.5%)	17 (34.7%)	2,086 (39.8%)
Weight gain of ≥ 10 kg from the age of 20	30 (27.8%)	15 (30.6%)	1,841 (35.1%)
State of biting and eating a meal			
Can bite and eat anything	91 (84.3%)	37 (75.5%)	4,487 (85.5%)
Feel difficulty in biting due to teeth problems	17 (15.7%)	12 (24.5%)	737 (14.1%)
Hardly bite	0 (0.0%)	0 (0.0%)	21 (0.4%)
Lifestyle improvement			
Already improving	25 (23.1%)	9 (18.4%)	1,401 (26.7%)
Willing to improve	62 (57.4%)	35 (71.4%)	2,848 (54.3%)
No intention to improve	21 (19.4%)	5 (10.2%)	996 (19.0%)
Willing to use opportunity to receive health instruction on lifestyle improvement	55 (50.9%)	30 (61.2%)	2,613 (49.8%)

*Abbreviations*: *FC* functional constipation, *IBS* irritable bowel syndrome, *IQR* interquartile range, *BMI* body mass index, *H. pylori Helicobacter pylori*

These characteristics of subjects with FC and IBS were consistent with our previous studies [15, 16].

### Upper gastrointestinal endoscopic findings associated with functional constipation or irritable bowel syndrome

Erosive gastritis, including raised erosion, depressive erosion, and corpus erosion (51.0%, 30.6%, 10.2%, and 18.4%, respectively) and duodenitis (24.5%) were the most common in subjects with IBS. In contrast, red streaks (48.1%) were the most common in subjects with FC (Table 2).

**Table 2:** Upper gastrointestinal endoscopic findings in subjects with functional constipation, irritable bowel syndrome, and controls

**Variables**	**FC**	**IBS**	**Controls**
Number of cases	108	49	5,245
Esophagus			
Hiatus hernia	8 (7.4%)	8 (16.3%)	727 (13.9%)
Reflux esophagitis (modified LA grade M–D)	12 (11.1%)	4 (8.2%)	767 (14.6%)
Stomach			
Erosive gastritis	17 (15.7%)	25 (51.0%)	611 (11.6%)
Raised erosion	5 (4.6%)	15 (30.6%)	371 (7.1%)
Depressive erosion	3 (2.8%)	5 (10.2%)	111 (2.1%)
Corpus erosion	9 (8.3%)	9 (18.4%)	153 (2.9%)
Red streaks	52 (48.1%)	15 (30.6%)	881 (16.8%)
Gastric fundic gland polyp	32 (29.6%)	14 (28.6%)	1,606 (30.6%)
Atrophic gastritis			
Open-type	27 (25.0%)	15 (30.6%)	1,615 (30.8%)
Closed-type	81 (75.0%)	34 (69.4%)	3,630 (69.2%)
Duodenum			
Duodenitis	4 (3.7%)	12 (24.5%)	317 (6.0%)

*Abbreviations FC* functional constipation, *IBS* irritable bowel syndrome, *LA* Los Angeles classification

Robust Poisson regression analyses adjusted for age, sex, *H. pylori* infection status, alcohol intake, and smoking habits showed a significant association between FC and corpus erosion (adjusted prevalence ratio [aPR], 2.93; 95% CI, 1.51–5.67; *p* < 0.01) and red streaks (aPR, 3.83; 95% CI, 2.53–5.79; *p* < 0.01) (Table 3). Adjusted robust Poisson regression analyses revealed that erosive gastritis (aPR, 8.46; 95% CI, 4.89–14.67; *p* < 0.01) and duodenitis (aPR, 7.28; 95% CI, 3.64–14.59; *p* < 0.01) were significantly associated with IBS. Even in separate analyses of erosive gastritis, raised erosion (aPR, 6.70; 95% CI, 3.66–12.26; *p* < 0.01), depressive erosion (aPR, 5.28; 95% CI, 2.19–12.71; *p* < 0.01), and corpus erosion (aPR, 6.92; 95% CI, 3.42–13.98; *p* < 0.01) were all significantly associated with IBS. Red streaks tended to be associated with IBS (aPR, 1.96; 95% CI, 1.00–3.83; *p* = 0.05), although it was not statistically significant (Table 4).

**Table 3:** Robust Poisson regression analyses of the upper gastrointestinal endoscopic findings associated with functional constipation

**Variables**	**Crude PR (95% CI)**	***p*-value**	**Adjusted PR (95% CI)**^a^	***p*-value**
Esophagus				
Hiatus hernia	0.50 (0.25–1.03)	0.06	0.66 (0.33–1.34)	0.25
Reflux esophagitis (modified LA grade M–D)	0.74 (0.41–1.34)	0.32	1.11 (0.61–2.02)	0.73
Stomach				
Erosive gastritis	1.36 (0.82–2.27)	0.24	1.59 (0.95–2.68)	0.08
Raised erosion	0.62 (0.26–1.52)	0.30	0.76 (0.31–1.87)	0.55
Depressive erosion	1.27 (0.41–3.94)	0.68	1.42 (0.45–4.50)	0.55
Corpus erosion	2.78 (1.43–5.41)	< 0.01	2.93 (1.51–5.67)	< 0.01
Red streaks	4.36 (3.01–6.32)	< 0.01	3.83 (2.53–5.79)	< 0.01
Gastric fundic gland polyp	0.96 (0.64–1.44)	0.83	0.81 (0.52–1.24)	0.33
Atrophic gastritis				
Open-type	0.87 (0.46–1.65)	0.66	0.95 (0.49–1.84)	0.88
Closed-type	1		1	
Duodenum				
Duodenitis	0.59 (0.22–1.58)	0.29	1.06 (0.40–2.82)	0.91

*Abbreviations PR* prevalence ratio, *CI* confidence interval, *LA* Los Angeles classification

^a^Adjusted for age, sex, *Helicobacter pylori* infection status, alcohol intake, and smoking habits

**Table 4:** Robust Poisson regression analyses of the upper gastrointestinal endoscopic findings associated with irritable bowel syndrome

**Variables**	**Crude PR (95% CI)**	***p*-value**	**Adjusted PR (95% CI)**^a^	***p*-value**
Esophagus				
Hiatus hernia	1.22 (0.58–2.60)	0.60	1.47 (0.67–3.23)	0.33
Reflux esophagitis (modified LA grade M–D)	0.52 (0.19–1.45)	0.22	0.59 (0.22–1.55)	0.28
Stomach				
Erosive gastritis	7.58 (4.35–13.18)	< 0.01	8.46 (4.89–14.67)	< 0.01
Raised erosion	5.65 (3.11–10.29)	< 0.01	6.70 (3.66–12.26)	< 0.01
Depressive erosion	5.05 (2.04–12.50)	< 0.01	5.28 (2.19–12.71)	< 0.01
Corpus erosion	6.88 (3.40–13.96)	< 0.01	6.92 (3.42–13.98)	< 0.01
Red streaks	2.07 (1.13–3.79)	0.02	1.96 (1.00–3.83)	0.05
Gastric fundic gland polyp	0.91 (0.49–1.68)	0.76	0.89 (0.46–1.72)	0.73
Atrophic gastritis				
Open-type	0.55 (0.17–1.78)	0.32	0.72 (0.19–2.65)	0.62
Closed-type	1		1	
Duodenum				
Duodenitis	4.94 (2.60–9.38)	< 0.01	7.28 (3.64–14.59)	< 0.01

*Abbreviations*: *PR* prevalence ratio, *CI* confidence interval, *LA* Los Angeles classification

^a^Adjusted for age, sex, *Helicobacter pylori* infection status, alcohol intake, and smoking habits

### Sensitivity analysis

We calculated the E-value for aPR and the corresponding lower limit of the 95% CI as a sensitivity analysis to evaluate robustness. In association with FC, the E-value was 5.31 (E-value for the lower limit of 95% CI = 2.39) for corpus erosion and 7.12 (E-value for the lower limit of 95% CI = 4.50) for red streaks. In association with IBS, the E-value was 16.40 (E-value for the lower limit of 95% CI = 9.25) for erosive gastritis, 3.33 (E-value for the lower limit of 95% CI = 1.00) for red streaks, and 14.04 (E-value for the lower limit of 95% CI = 6.74) for duodenitis.

### Gastrointestinal and psychological symptoms in subjects with functional constipation, those with irritable bowel syndrome, and controls

**Fig. 3:**
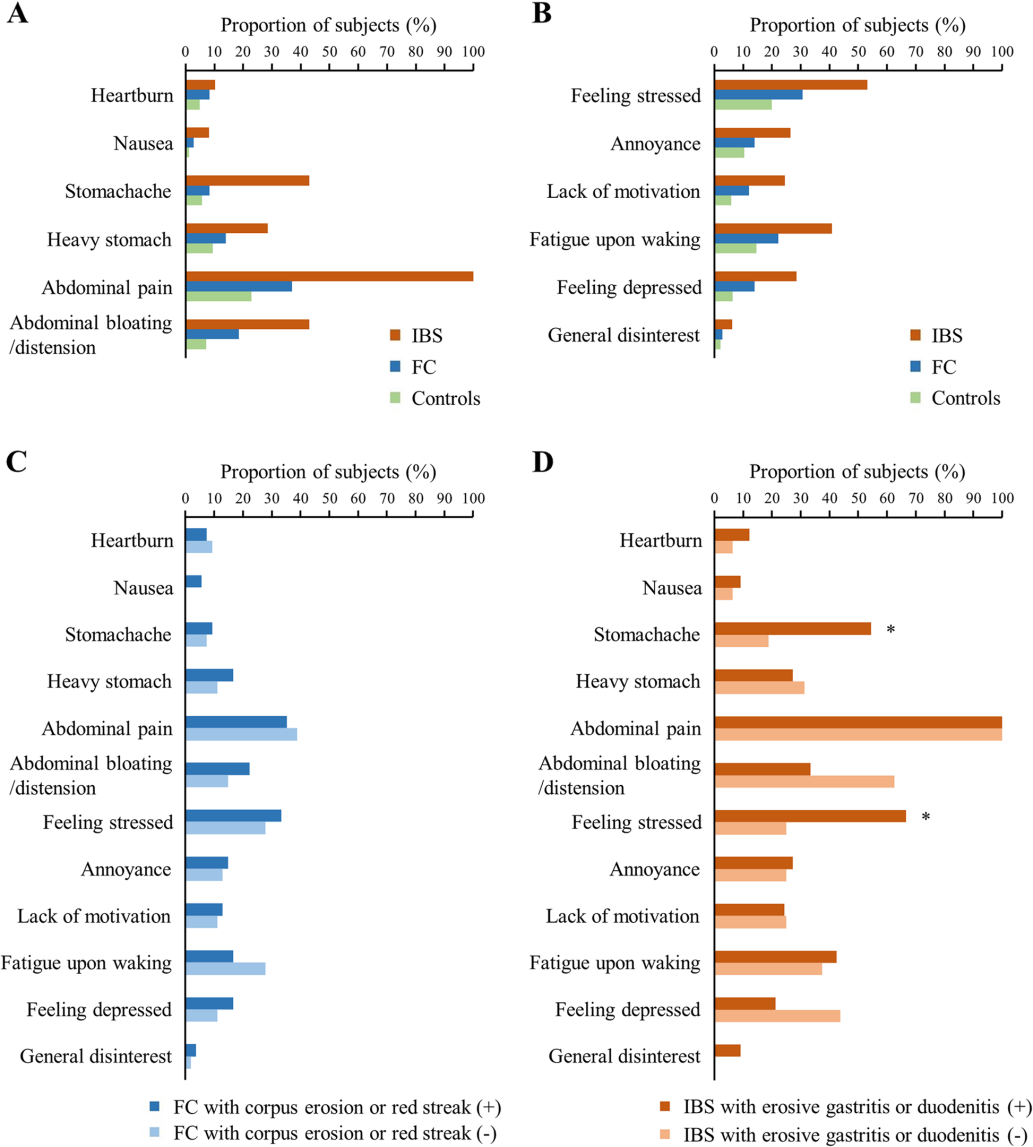
Gastrointestinal and psychological symptoms in subjects with functional constipation, irritable bowel syndrome, and controls. **A** Gastrointestinal symptoms, **B** Psychological symptoms, **C** Symptoms with or without corpus erosion or red streaks in subjects with functional constipation, **D** Symptoms with or without erosive gastritis or duodenitis in subjects with irritable bowel syndrome; Abbreviations: IBS, irritable bowel syndrome; FC, functional constipation; ^*^P < 0.05

Subjects with IBS were the most to complain of both upper GI symptoms, such as heartburn, nausea, stomachache, and heavy stomach (10.2%, 8.2%, 42.9%, and 28.6%, respectively), and lower GI symptoms, such as abdominal pain and abdominal bloating/distension (100.0% and 42.9%, respectively), followed by those with FC and controls (Fig. 3A). Furthermore, subjects with IBS were the most to complain of psychological symptoms, such as feeling stressed, annoyance, lack of motivation, fatigue upon waking, and feeling depressed (53.1%, 26.5%, 24.5%, 40.8%, and 28.6%, respectively), followed by those with FC and controls (Fig. 3B).

### The relationship between symptoms and upper gastrointestinal endoscopic findings associated with functional constipation or irritable bowel syndrome

We further investigated whether GI and psychological symptoms differed with or without corpus erosion or red streaks in subjects with FC, and the symptoms were not remarkably different depending on the presence of corpus erosion or red streaks (Fig. 3C). We then investigated whether GI and psychological symptoms differed with or without erosive gastritis or duodenitis in subjects with IBS. IBS subjects with erosive gastritis or duodenitis had significantly more complaints of stomachache and feeling stressed than those without erosive gastritis or duodenitis (54.5% vs. 18.8%; *p* = 0.03 and 66.7% vs. 25.0%; *p* = 0.01) (Fig. 3D).

## Discussion

It was notably identified in this study that erosive gastritis, red streaks, and duodenitis, which were the upper GI endoscopic findings considered to be caused by gastric acid or other etiologies, were associated with FC or IBS, although they are classified as bowel disorders in DGBI, according to the Rome IV criteria. No significant differences in the *H. pylori* infection status were present among the subjects with FC, IBS, and controls, and variables of age, sex, *H. pylori* infection status, alcohol intake, and smoking habits that may influence these endoscopic findings were adjusted in multivariate analyses. Moreover, we excluded participants who were taking LDA or NSAIDs that could cause erosive gastritis, red streaks, and duodenitis and those who were taking acid secretion inhibitors that could heal the lesions that would have appeared with FC or IBS. Thus, it is suggested that these endoscopic findings are closely associated with FC or IBS.

Red streaks were significantly associated with FC and had a tendency to be associated with IBS. Corpus erosion was significantly associated with both FC and IBS. Therefore, red streaks and corpus erosion may be common upper GI findings in subjects with FC and IBS. Although the etiology of the red streaks is unknown, it is considered that functional abnormality in GI tract is possible. Red streaks appear on the longitudinal folds, and mucosa on the ridge of folds is highly exposed to abnormal gastric peristalsis and gastric acid. Red streaks may be caused by local congestion on the ridge of folds during peristalsis as superficial blood vessels are observed, while inflammatory cell infiltration is not observed in the histopathological findings of the lesion [17]. Motility disturbance is the major pathophysiology of FC, and autonomic dysfunction, degeneration of the myenteric plexus, and decreased levels of neurotransmitters, such as serotonin, substance P, nitric oxide, vasoactive intestinal peptide, and calcitonin gene-related peptide, have been observed in patients with slow-transit constipation [6, 18–24]. These factors involved in FC can also cause motility disturbance in IBS, affecting not only the lower GI motility but also gastric motility. Corpus erosion is found in both *H. pylori*-positive and negative mucosa, and sometimes appears as longitudinal or linear erosion, accompanied by red streaks in the corpus. Therefore, it is reasonable to assume that corpus erosion is also associated with FC and IBS. In the lower GI tract, Gupta et al. reported that constipation did not show any increased risk of significant findings on colonoscopy from a retrospective review of the national endoscopic database [25]. To our knowledge, this is the first report demonstrating the upper GI findings of FC.

Meanwhile, duodenitis and other components of erosive gastritis, such as raised erosion and depressive erosion, were also significantly associated with IBS. Consistent with our results, Loosen et al. reported that the most common overlapping GI diagnoses with IBS are intestinal infectious diseases, gastritis/duodenitis, esophageal diseases, FD, and other diseases of the stomach and duodenum in subjects with IBS using the disease analyzer database in Germany [26]. There are reports demonstrating the dysbiosis of mucosa-associated microbiota in duodenum in subjects with IBS [27, 28], suggesting that altered duodenal microbiota can be one of the causes of duodenitis in subjects with IBS.

Furthermore, we examined the symptoms in subjects with FC and IBS. Although FC and IBS are classified as bowel disorders, a substantial proportion of subjects with both disorders had upper GI symptoms. Subjects with FC complained more of a variety of GI and psychological symptoms than control group, suggesting that this disease is one of the DGBI. Similarly, Wald et al. reported that the psychological distress score was high in patients with normal-transit constipation [29]. Considering that there were no significant differences in GI and psychological symptoms between FC subjects with corpus erosion or red streaks and those without corpus erosion or red streaks in this study, these endoscopic findings were not responsible for symptoms in subjects with FC, and endoscopic findings and symptoms may independently originate from motility disturbance or psychological distress. On the other hand, subjects with IBS more complained of GI and psychological symptoms than those with FC and controls. Consistent with our results, it has been reported that subjects with IBS frequently have upper GI and mental symptoms in addition to lower GI symptoms [7] and GI symptoms are generally more severe in subjects with IBS than in those with constipation [30]. Among the upper GI symptoms, stomachache was more common in subjects with erosive gastritis or duodenitis, suggesting that low-grade inflammation and immunological alterations may manifest as stomachache in IBS subjects with visceral hypersensitivity [31, 32]. Visceral hypersensitivity includes allodynia and hyperalgesia, and subjects with IBS experience strong visceral pain even with stimuli that do not cause pain or only mild pain in healthy individuals [33]. Altered central nervous system or brain function has been demonstrated in patients with IBS using brain imaging [34], and it may partially explain the symptoms in subjects with IBS. Lee et al. reported that patients with IBS had higher level of psychological symptoms, such as depression or anxiety [35], and those with more severe symptoms might meet the diagnostic criteria for psychiatric comorbidities, such as depressive or anxiety disorders. Among the psychological symptoms in subjects with IBS, feeling stressed was more common in subjects with erosive gastritis or duodenitis. Strong stress could be a cause of erosive gastritis and duodenitis in subjects with IBS since it has been shown that stress increases the level of gastric acid and decreases the motility in animal studies [36, 37]. In addition, subjects with IBS had more disturbed lifestyle habits such as inability to get enough rest by sleep in this study, which is consistent with previous reports that noted the prevalence of sleep disorders to be higher in patients with IBS [38, 39]. These symptoms and disturbed lifestyle habits may result in impairment of quality of life in subjects with IBS [40].

This study had some limitations. First, it was conducted at a single medical check-up institution, and the characteristics of the subjects might have been biased. The participants who underwent a medical check-up were highly conscious of their health, and their overall health status was generally good. Moreover, very elderly and very young individuals usually do not undergo medical check-ups. Therefore, the prevalence of FC and IBS in this study was lower than that in outpatients with FC and IBS in a hospital setting. Second, the questionnaire used in this study was based on self-reported measures by participants, and issues such as missed reporting and providing inaccurate information were unavoidable. Third, IBS subtypes cannot be diagnosed in this study since a questionnaire used for diagnosing FC and IBS did not inquire about IBS subtyping. Fourth, accurate *H. pylori* infection status is difficult to be determined because only anti-*H. pylori* antibody is measured during a single medical check-up. Although anti-*H. pylori* antibody ≥ 10 U/mL without eradication therapy generally indicates current *H. pylori* infection, it is ideal to perform more than two types of examinations to increase the accuracy. However, our study has strengths in that the cross-sectional survey was conducted prospectively for all participants to avoid selection bias and FC and IBS were strictly diagnosed baesd on the latest international standard Rome IV criteria. This large-scale survey could accurately assess the upper GI endoscopic features of FC and IBS in subjects during a medical check-up.

## Conclusions

In conclusion, in the upper GI endoscopic findings, corpus erosion and red streaks were significantly associated with FC, and erosive gastritis and duodenitis were significantly associated with IBS. Red streaks were also possibly associated with IBS. A substantial proportion of subjects with FC and IBS had a variety of GI and psychological symptoms, and IBS subjects with erosive gastritis or duodenitis significantly complained of stomachache and feeling stressed. These findings suggest that some subjects with these minor abnormalities in the upper GI endoscopy in addition to troublesome symptoms may have FC or IBS. Therefore, we should not overlook these subjects during a medical check-up.

## Data Availability

The datasets used and analyzed during the current study are available from the corresponding author on reasonable request.

## Abbreviations

GI: Gastrointestinal
DGBI: Disorders of gut–brain interaction
FC: Functional constipation
IBS: Irritable bowel syndrome
FD: Functional dyspepsia
LDA: Low-dose aspirin
NSAIDs: Nonsteroidal anti-inflammatory drugs
IQR: Interquartile range
CI: Confidence interval
aPR: Adjusted prevalence ratio
